# Extraction of Illegal Dyes from Red Chili Peppers with Cholinium-Based Deep Eutectic Solvents

**DOI:** 10.1155/2017/2753752

**Published:** 2017-07-31

**Authors:** Shuqiang Zhu, Dongling Liu, Xinyue Zhu, Along Su, Haixia Zhang

**Affiliations:** ^1^Key Laboratory of Nonferrous Metal Chemistry and Resources Utilization of Gansu Province and College of Chemistry and Chemical Engineering, Lanzhou University, Lanzhou 730000, China; ^2^Gansu Food Inspection and Research Institute, Lanzhou 730030, China; ^3^Gansu Key Laboratory of Traditional Chinese Medicine Quality and Standard, Gansu University of Chinese Medicine, Lanzhou 730000, China

## Abstract

Deep eutectic solvents (DESs) as a new kind of green solvents have been used to extract bioactive compounds but there are few applications in extracting chrysoidine dyes. In this study, we developed an ultrasonic-assisted extraction method with choline chloride/hydrogen bond donor (ChCl/HBD) DES for the extraction of chrysoidine G (COG), astrazon orange G (AOG), and astrazon orange R (AOR) in food samples. Some experimental parameters, such as extraction time, raw material/solvent ratio, and temperature, were evaluated and optimized as follows: the ratio of ChCl/HBD, 1 : 2 (v/v); the ratio of sample/DES, 1 : 10 (g/mL); extraction time, 20 min; extraction temperature, 50°C. Under the optimized conditions, the limits of detection (*μ*g/mL) were 0.10 for COG and 0.06 for AOG and AOR. The relative standard deviations were in the range of 1.2–2.1%. The recoveries of the three dyes were in the range of 80.2–105.0%. By comparing with other commonly used solvents for extracting chrysoidine dyes, the advantages of DESs proved them to be potential extraction solvents for chrysoidine G, astrazon orange G, and astrazon orange R in foods.

## 1. Introduction

Food colorants are often added to improve color appearance and to promote sales in food [1]. As an attractive group of food additives, their use range and dosage are restricted strictly in many countries. In China, the use of synthetic colorants in foods is strictly controlled by Directive GB 2760-2014 [2] of the Ministry of Health [3]. In recent years, although natural food colorants have become more and more popular with consumer, synthetic colorants are still used illegally due to their low costs, high effectiveness, and good stability in many foods [4, 5]. Chrysoidine, a type of industrial azoic dye, is a kind of illegal additive banned in China [2, 6, 7] which could cause acute and chronic toxicity to mammals administrated by oral or skin route or inhaled [8] and has been confirmed to be genotoxic, mutagenic, and carcinogenic [9–11]. Unfortunately, chrysoidine has been reported in some red hot chili peppers in China [12].

Several analytical methods for the determination of dyes have been proposed, including high performance liquid chromatography UV (HPLC-UV) [13], liquid chromatography coupled to mass spectrometry (LC-MS) [14, 15], and enzyme linked immunosorbent assay (ELISA) with newly developed polyclonal antibodies [16]. In all the analytical methods, conventional organic solvents such as chloroform and ethyl acetate are widely used in the extraction of chrysoidine dyes from food samples.

To overcome the limitations of toxicity of organic solvents, deep eutectic solvents (DESs), as a new kind of green solvents, have emerged [17]. DES is generally composed of two or three nontoxic components, forming eutectic mixture with a much lower melting point than either of the individual components [18]. In general, one of the components is choline chloride (ChCl) and another component is hydrogen bond donor (HBD) containing functional groups of carboxylic acids, urea, or polyols [19, 20]. There have been a great number of studies on dissolution and separation in using DESs, such as CO_2_ solubility [21], dissolution of metal oxides [22] and drug [23], and purification of biodiesel [24]. DESs are capable of donating or accepting electrons or protons to form hydrogen bonds, which gives them excellent dissolution properties and potential for extraction [18, 25]. Up to now, no one has reported the application of DES for the extraction of COG, AOG, and AOR in food samples.

In this study, a series of DESs mixing ChCl and HBDs at different ratios were used to extract COG, AOG, and AOR from red hot chili peppers. Using the optimal system composition, other experimental parameters such as sample ratio, ultrasonic power, temperature, and time were optimized systematically. HPLC with UV detector was chosen as an analytical apparatus.

## 2. Methods and Materials

### 2.1. Chemicals and Materials

COG, AOG, and AOR (98%) were obtained from Sigma (St. Louis, MO, USA). ChCl (>98.0%), ethylene glycol (>99.0%), glycerol (>99.0%), 1,2-butanediol (>98.0%), 1,3-butanediol (>99.0%), and 1,4-butanediol (>99.0%) were purchased from Sinopharm Chemical Reagent Co. Ltd. (Shanghai, China). Methanol and acetonitrile of HPLC grade were supplied by Merck Chemical Co. Ltd. (Darmstadt, Germany). All the other solvents used in the experiment were of analytical grade. Ultrapure water was obtained from the Milli-Q system (Millipore, Bedford, MA, USA). All the samples were passed through a filter (Nylon, 0.45 *μ*m, MN, Germany) before HPLC-UV analysis.

### 2.2. Preparation of DES

DESs were prepared by heating ChCl and HBDs to 80°C with constant stirring until a homogeneous liquid was formed. ChCl was mixed with each HBD at the molar ratios (1 : 3), as shown in Table 1.

### 2.3. Extraction Procedure

Dried red hot chili peppers powder (0.10 g) was spiked with 5 *μ*g of COG, AOG, and AOR, respectively, and was extracted with 1.0 mL of the DES by the help of ultrasonic irradiation (75 W, 20 min). The turbid solutions obtained were centrifuged at 12,000 rpm for 5 min. The supernatant (500 *μ*L) was mixed with an equal volume of methanol and filtered (0.45 *μ*m) before being analyzed by HPLC-UV. Each group experiment was repeated three times.

### 2.4. HPLC Instrumentation

A Waters e2695 four-solvent gradient pump and a Waters UV2487 detector (Waters Co., USA) were used for HPLC-UV analysis. Separations were done using an ODS-C_18_ column (250 mm × 4.6 mm i.d., 5 *μ*m, Krosmail Co., Sweden). The mobile phase, methanol and 10 mmol/L of ammonium acetate aqueous solution (70 : 30, v/v), was used as the isocratic elution at room temperature. The flow-rate, UV wavelength, and injection volume were set to 1.0 mL/min, 485 nm, and 10.0 *μ*L, respectively.

## 3. Results and Discussion

### 3.1. Selection of DESs

**Figure 1:**
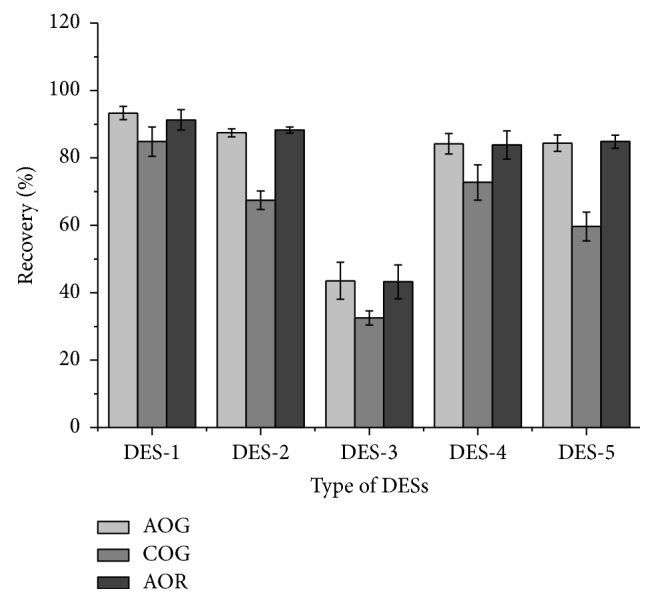
Effect of different type of DES on the recoveries of COG, AOG, and AOR. Molar ratio of ChCl to HBD: 1/3, sample/liquid: 0.1 g/mL, temperature: 30.0°C, time: 20 min, and ultrasonic power: 75 W.

The choice of extraction method and type of DESs were very important for extracting COG, AOG, and AOR from red hot chili peppers. As shown in Figure 1, the recovery of COG, AOG, and AOR extracted using DES-1 was optimum compared to the other DESs. Except DES-3, the other DESs offered similar recovery results for AOG and AOR. COG recovery was affected the most by the different kinds of HBD. Glycerol (in DES-3) was not found to be a suitable HBD for the extraction of these dyes. The three hydroxyl groups in the glycerol molecule exhibit stereospecific blockades with each other, which was not beneficial to forming of hydrogen bonds with the dyes.

The physicochemical properties, such as viscosity, surface tension, and polarity, have a significant effect on the extraction efficiency of DES. The viscosity and surface tension of DES-1 are the lowest among the five DESs tested and the polarity is relatively high [19]. The low viscosity of DES brings about high diffusivity [26, 27] which can improve the extraction efficiency of the three dyes. In consideration of similarity-intermiscibility theory, the polarity of DES-1 appears to be more similar to those of AOR (log P 6.82) and AOG (log P 5.14) than those of COG (log P 2.71, calculated by Discovery Studios), which is responsible for its high extractability. However, the extraction efficiency of DES-3 was much lower than that of other DESs because of its high viscosity and surface tension.

### 3.2. Effect of ChCl/HBD Molar Ratio

**Figure 2:**
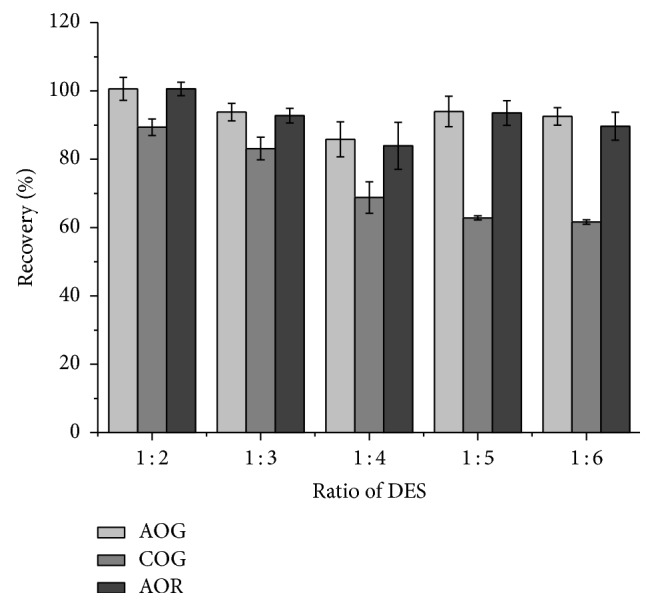
Effects of ChCl/HBD molar ratios on the recoveries of dyes. Sample/liquid: 0.1 g/mL, temperature: 30.0°C, time: 20 min, and ultrasonic power: 75 W.

Different ChCl/HBD ratios (1 : 2, 1 : 3, 1 : 4, 1 : 5, and 1 : 6) in DES of ChCl and ethyl glycol (DES-1) were evaluated. As shown in Figure 2, the AOG and AOR were almost entirely extracted in the DES with different ratios except in the DES of 1 : 4. The recovery of COG decreased with the increasing of ethyl glycol in DES. Finally the DES with the ratio of 1 : 2 was chosen for the further experiments, named DES-1-2.

**Figure 3:**
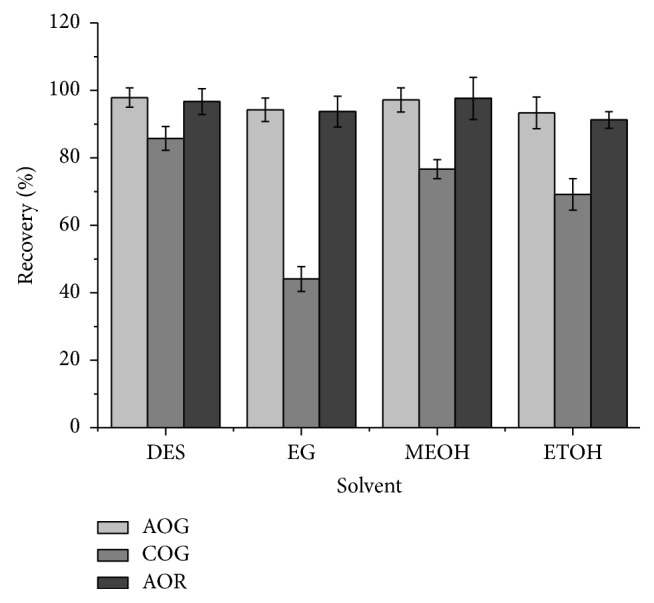
Effect of different solvents on the recoveries of dyes. DES: DES-1-2, sample/liquid: 0.1 g/mL, temperature: 30°C, time: 20 min, and ultrasonic power: 75 W.

To confirm the extraction efficiency of DES-1-2, several organic solvents such as ethylene glycol (EG), methanol (MEOH), and ethanol (ETOH) were applied to extract the three dyes. As shown in Figure 3, although the recoveries of both AOG and AOR extracted by DES-1-2 and the three organic solvents ran up to 93%, the recovery of COG extracted by DES-1-2 was the highest.

### 3.3. Effect of Sample/Liquid Ratio

**Figure 4:**
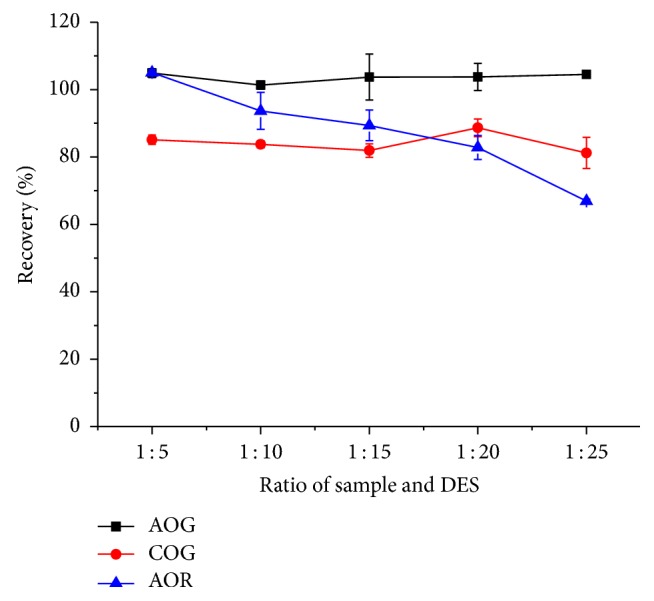
Effect of ratio of sample/DES-1-2 on the recoveries of COG, AOG, and AOR. Temperature: 30°C, time: 20 min, and ultrasonic power: 75 W.

The sample/DES-1-2 ratio was explored. A range of sample/liquid ratios (1 : 5, 1 : 10, 1 : 15, 1 : 20, and 1 : 25, (g/mL)) were adopted to achieve the optimum ratios. As shown in Figure 4, the recoveries of the COG, AOG, and AOR were within 83.7 to 101.4%, when the sample/liquid ratio was 1 : 10 or 1 : 5. However, the higher sample/liquid ratio than 1/10 (g/mL) did not significantly affect the recoveries of the COG and AOG, whereas the recoveries of the AOR decreased. Finally, the sample/liquid ratio 1/10 was chosen because the ratio 1/5 resulted in much less liquid to operate.

### 3.4. Effect of Temperature

**Figure 5:**
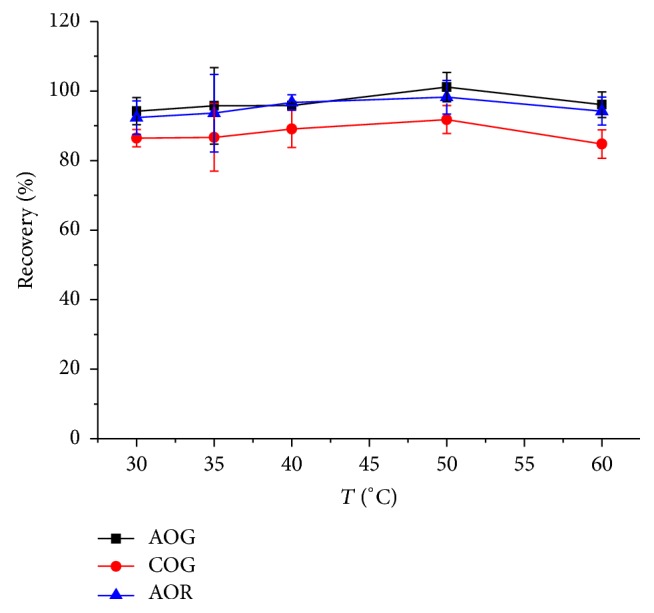
Effect of temperature on the recoveries of dyes. Sample/DES-1-2: 0.1 g/mL, time: 20 min, and ultrasonic power: 75 W.

Temperature can affect the physicochemical properties of DES, so its effect on the extraction was studied. Generally, high temperatures were accompanied with lower viscosity and surface tension. As shown in Figure 5, the recoveries of the dyes gently increased until the temperature increased to 50°C, but there was a little decrease when the temperature was above 50°C. Therefore, 50°C was selected as the appropriate temperature.

### 3.5. Effect of Extraction Time

**Figure 6:**
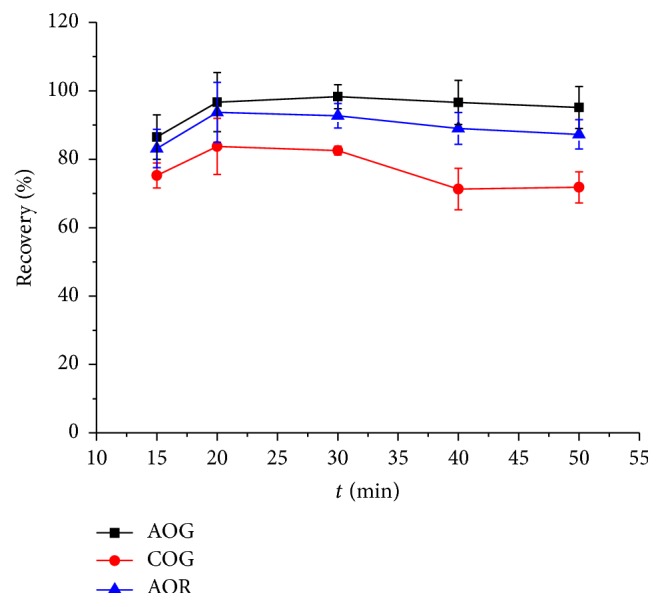
Effect of extraction time on the recoveries of the three dyes. Sample/DES-1-2: 0.1 g/mL, temperature: 50.0°C, and ultrasonic power: 75 W.

The ultrasonic time from 15 to 50 min was also optimized for the extraction of these dyes. In Figure 6, the largest recoveries of AOG and AOR were achieved after being treated for 20 min and underwent no change even for longer time. For COG, a period longer than 30 min was no help to the extraction. Hence 20 min was adopted.

### 3.6. Validation of the Proposed Method

**Figure 7:**
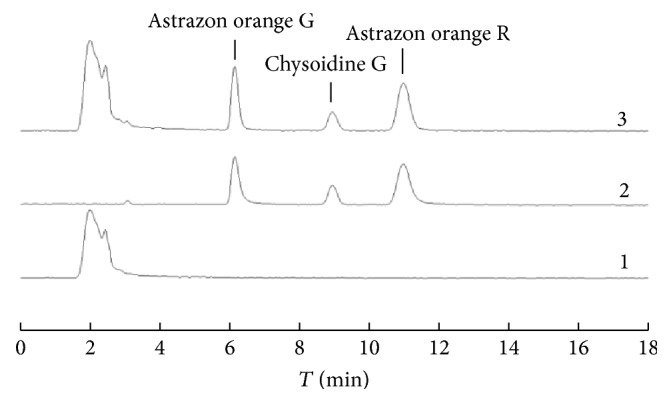
Chromatograms of the red hot chili peppers (curve 1), the standard of three dyes (each 2 *µ*g/mL, curve 2), and the red hot chili peppers spiked standard solution (each 2.5 *µ*g/mL, curve 3).

The optimum conditions of ChCl-based DES-1-2 as extractant in our experiments were as follows: the ratio of ChCl/HBD, 1 : 2; sample/DES-1-2 ratio, 1 : 10 (g/mL); extraction time, 20 min; extraction temperature, 50°C. Under the above conditions, we tested the linearity, precision, detection limits, and other characteristics for the method (Table 2). A series of the three dyes with different concentrations in the range of 0.5–20.0 *μ*g/mL were prepared to make calibration curves with correlation coefficients larger than 0.9997. The precision tests were done by carrying out five parallel experiments for each compound: the relative standard deviation (RSD) values, the limit of detection (LOD) values based on the chromatographic signal to the baseline noise ratio (*S*/*N* = 3), and the limit of quantification (LOQ) based on *S*/*N* = 10.  Figure 7 showed the chromatograms of the blank red hot chili peppers (curve 1), the standards (2 *μ*g/mL, curve 2), and the red hot chili peppers spiked standard solution (2.5 *μ*g/mL, curve 3).

### 3.7. Application

The optimized method was used to determine the target compounds in different foods (red hot chili peppers, Chinese red peppers, and bean curd stick) from a local market. No dyes were found. Then the dyes were added to the samples and the recoveries were calculated, as shown in Table 3. The experimental results confirmed that the method was validated for the determination of COG, AOG, and AOR in real samples.

## 4. Conclusions

In this work, we developed a green and efficient extraction method, involving ultrasonic-assisted extraction of food samples with ChCl/Ethyl glycol (1 : 2) DES for the extraction of COG, AOG, and AOR. The experimental results showed the DES as extractant could offer the satisfying recovery with less organic solvent consumed with the simple operation. The method could be used successfully for the analysis of illegal additive dyes (COG, AOG, and AOR) in solid food.

## Figures and Tables

**Table 1:** DESs synthesized in the work.

Product	Salt	HBD	Mole ratio (ChCl : HBD)
DES1	ChCl	Ethyl glycol	1 : 3
DES2	ChCl	1,2-Butanediol	1 : 3
DES3	ChCl	Glycerol	1 : 3
DES4	ChCl	1,3-Butanediol	1 : 3
DES5	ChCl	1,4-Butanediol	1 : 3

**Table 2:** Parameters of analytical performance of the proposed method (*n* = 3).

Analyte	*R* ^2^	RSD (%)	Spiked (*µ*g)	Detected (*µ*g)	Recovery (%)	LOD (*µ*g/mL)	LOQ (*µ*g/mL)
AOG	0.9997	1.2	0.5	0.46	91.5 ± 4.2	0.06	0.21
			5.0	4.71	94.3 ± 2.6		
			10.0	10.51	105.0 ± 1.2		
COG	0.9998	2.1	0.5	0.40	80.2 ± 3.1	0.10	0.33
			5.0	4.26	85.2 ± 2.8		
			10.0	9.20	92.0 ± 1.9		
AOR	0.9998	1.3	0.5	0.43	86.8 ± 3.4	0.06	0.29
			5.0	4.68	93.6 ± 1.8		
			10.0	10.0	100.0 ± 2.3		

**Table 3:** Recoveries of the dyes in different food samples with this method (*n* = 3).

Products	Compounds	Spiked (*µ*g)	Detected (*µ*g)	Recovery (%)
Red hot chili peppers	AOG		2.4	95.6 ± 2.7
	COG	2.5	2.2	90.0 ± 1.3
	AOR		2.3	91.2 ± 4.4
Chinese red peppers	AOG		2.3	91.2 ± 2.3
	COG	2.5	2.4	96.0 ± 2.5
	AOR		2.3	90.4 ± 2.7
Bean curd stick	AOG		2.5	100.8 ± 1.1
	COG	2.5	2.4	97.6 ± 0.4
	AOR		2.5	99.6 ± 2.0
